# The L730V/I RET roof mutations display different activities toward pralsetinib and selpercatinib

**DOI:** 10.1038/s41698-021-00188-x

**Published:** 2021-06-07

**Authors:** Tao Shen, Xueqing Hu, Xuan Liu, Vivek Subbiah, Blaine H. M. Mooers, Jie Wu

**Affiliations:** 1 Peggy and Charles Stephenson Cancer Center, University of Oklahoma Health Sciences Center, Oklahoma City, OK USA; 2 Department of Pathology, University of Oklahoma Health Sciences Center, Oklahoma City, OK USA; 3 Department of Investigational Cancer Therapeutics, Division of Cancer Medicine, the University of Texas MD Anderson Cancer Center, Houston, TX USA; 4 Department of Biochemistry and Molecular Biology, University of Oklahoma Health Sciences Center, Oklahoma City, OK USA; 5 Laboratory of Biomolecular Structure and Function, University of Oklahoma Health Sciences Center, Oklahoma City, OK USA

**Keywords:** Cancer, Molecular medicine, Targeted therapies, Phenotypic screening, Lung cancer

## Abstract

Recently Food and Drug Administration (FDA)-approved pralsetinib (BLU-667) and selpercatinib (LOXO-292) are RET-selective protein tyrosine kinase inhibitors for treating RET-altered cancers, but whether they have distinct activity was unknown. The L730V/I mutations at the roof of the solvent-front site of the RET kinase were identified as strongly resistant to pralsetinib but not to selpercatinib. Selpercatinib effectively inhibited these mutants and the KIF5B-RET(L730V/I) oncogene-driven tumors.

Acquired resistance usually develops in protein tyrosine kinase-targeted cancer therapies, necessitating the discovery of secondary drugs that can suppress the resistant mechanisms for continuing tumor control^1^. A mechanism of acquired resistance to protein tyrosine kinase inhibitors (TKIs) is secondary mutations in the targeted kinases^1–3^. Second and third generations of TKIs have been used successfully to circumvent target mutation-associated resistance to earlier generations of EGFR, ALK, or ROS1 TKIs in non-small cell lung cancer (NSCLC)^1–3^. Thus, using different TKIs sequentially is a proven approach in targeted cancer therapy. This approach requires finding secondary drugs capable of inhibiting acquired mutations that cause resistance to the first drug used in the treatment.

Oncogenic RET kinase is a target for cancer therapy^4^. Selpercatinib^5–7^ and pralsetinib^8,9^ are potent and selective RET TKIs recently approved by the United States Food and Drug Administration as RET-targeted cancer drugs. While selpercatinib and pralsetinib had high response rates and the responses were more durable than multikinase TKIs in RET-altered cancers^5,6,8,9^, several recent studies have identified acquired selpercatinib-resistant RET mutations located at the floor of the solvent-front (G810C/S/R), the hinge (Y806C/N), and the β2 strand (V738A) of the RET ATP-binding site^10–12^ in addition to target-bypass mechanisms^11,13,14^. Among these mutations, the G810C/S/R mutations displayed the strongest resistance^12^ and were observed more often in patients whose tumors developed resistance to selpercatinib. Selpercatinib-resistant RET mutations identified so far were cross-resistant to pralsetinib^12^.

In this study, we identified the L730V/I RET mutations as being strongly resistant to pralsetinib but not to selpercatinib. These mutations differ from the G810 substitutions by being located at the roof of the solvent-front region. Crystal structures of the protein-drug complexes suggest that the L730V/I mutations introduce stronger steric clashes with pralsetinib. While pralsetinib could not inhibit the growth of xenograft tumors derived from BaF3/KIF5B-RET(L730V/I) cells, selpercatinib effectively inhibited these tumors in the animal model.

To identify pralsetinib-resistant RET mutations, we screened a random mutation pool of KIF5B-RET cDNA in the RET kinase-dependent BaF3/KIF5B-RET cells for pralsetinib resistance. Seven mutations in the RET kinase domain were identified from 54 pralsetinib-resistant cell lines (Table 1). Four of these mutations (V738A, Y806C/N, and G810S) were also identified previously as selpercatinib-resistant RET mutations^12^. Two of the new pralsetinib-resistant mutations (L730V/I) were located at the roof of the solvent front, whereas the third new mutation (E732K) was located in the Gly-rich loop. Cross-profiling of pralsetinib and selpercatinib for these three mutants in BaF3/KIF5B-RET cells (Supplementary Fig. 1) showed that the L730V/I mutants had 58- to 61-fold increased IC_50_s for pralsetinib (Table 1, Fig. 1a), which were greater than the G810S mutant (40-folds) (Table 1) and similar to the G810C mutant (70-folds)^12^. Strikingly, L730V/I had little effect on the sensitivity of selpercatinib with 4- to 7-fold higher IC_50_s, which were similar to that of the V804M gatekeeper mutation (IC_50_: 56.4 ± 1.9 nM, 7-folds) (Table 1, Fig. 1a). Because V804M-positive RET-altered cancer patients responded to selpercatinib^5,6^, L730V/I mutations were considered sensitive to selpercatinib. Immunoblotting assays of RET kinase activity and apoptosis of BaF3/KIF5B-RET and L730V/I mutant cells showed that pralsetinib could not inhibit KIF5B-RET phosphorylation or induce apoptosis of the mutant cells. In contrast, selpercatinib had comparable activity in inhibiting KIF5B-RET and KIF5B-RET(L730V/I) phosphorylation and inducing apoptosis in these cells (Fig. 1b, Supplementary Fig. 2).

**Table 1:** Identification and characterization of pralsetinib-resistant RET mutations.

Mutant	Pralsetinib (nM)			Subtotal	IC_50_ (nM) (fold change: mutant/wt)	
	120	150	300		Pralsetinib	Selpercatinib
L730I	3	3	4	10	592 ± 11.1 (61)	31.6 ± 9.7 (4)
L730V	0	0	1	1	568 ± 8.0 (58)	53.0 ± 1.2 (7)
E732K	1	1	0	2	162.3 ± 5.3 (17)	73.8 ± 2.1 (9)
V738A	0	2	0	2	177.5 ± 6.7^a^ (18)	238.8 ± 7.2^a^ (30)
Y806C	4	1	0	5	295.8 ± 10.7^a^ (30)	174.4 ± 5.4^a^ (22)
Y806N	9	10	1	20	292.5 ± 5.9^a^ (30)	149.8 ± 6.3^a^ (19)
G810S	6	4	4	14	390.6 ± 10.8^a^ (40)	880.2 ± 25.6^a^ (110)
WT	n/a			n/a	9.7 ± 0.6 (1)	8.0 ± 0.4 (1)

^a^Previously reported values^12^.

**Fig. 1:**
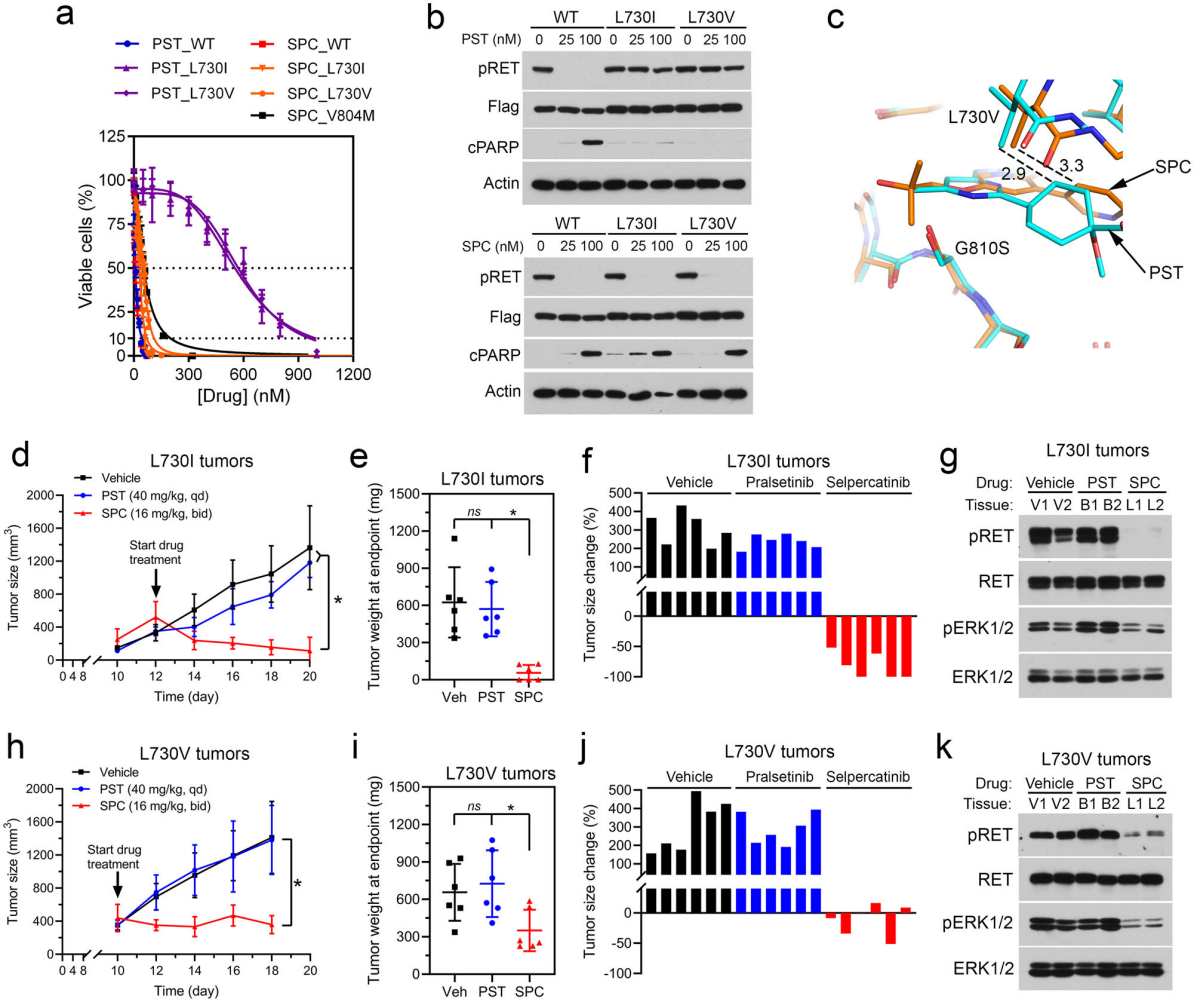
L730V/I display different sensitivity to pralsetinib and selpercatinib. **a** BaF3 cells containing KIF5B-RET or KIF5B-RET with indicated mutations were treated with pralsetinib or selpercatinib for 3 days, and viable cells were determined. Data points are means and s.d. **b** Cell lysates were analyzed by immunoblotting with indicated antibodies. **c** The vicinity of L730 and G810 of RET kinase. The carbon atoms of the pralsetinib complex (PDB: 7JU5) are colored cyan, and those of the selpercatinib complex (PDB: 7JU6) are colored orange. The sidechain at site 730 has been changed to a valine, modeled in its most favorable state in the absence of the drugs. G810 has been changed to serine. **d**–**g** data from L730I tumors. **d** BaF3/KIF5B-RET(L730I) xenograft tumors were treated with pralsetinib, selpercatinib, or vehicle. Tumor sizes were measured on the indicated days. The data shown are the mean ± s.d. **e** Tumor weight at the endpoint. The error bars shown are mean ± s.d. *p < 0.05. ns, *p* > 0.05. **f** Waterfall plot of tumor size change at the endpoint from the baseline (prior to drug treatment). Each bar corresponds to a tumor in each mouse (6 per group). **g** Tissue samples from two different mice in each group were analyzed by immunoblotting with the indicated antibodies. PST pralsetinib, SPC selpercatinib. **h**–**k** data from the BaF3/KIF5B-RET(L730V) tumors. The experiment was performed in parallel to the BaF3/KIF5B-RET(L730I) tumors as described above for the (**d**–**g**) panels.

In the co-crystal structures of pralsetinib-RET (PDB code: 7JU5) and selpercatinib-RET (PDB code: 7JU6) complexes^12^, the replacement of a C-gamma branched side chain with a C-beta branched side chain at site 730 leads to greater steric clashes between site V730 or I730 and pralsetinib than with selpercatinib (Fig. 1c). Pralsetinib has a six-membered methylaminopyrimidine ring where selpercatinib has a 2-hydroxy-2-methylpropoxy chain. The smaller bulk of the latter group reduces the probability of clashes with the beta-branched side chains of V730 and I730. Also, the cyclohexane ring in the pralsetinib is 0.4 Å closer to the beta carbon atoms at site 730 than the pyridine ring in the selpercatinib thereby leading to additional clashes (Fig. 1c). In contrast, the C-lobe solvent front G810S mutant had a much higher impact on selpercatinib (Table 1). The modeled serine sidechain at site 810 would more easily clash with the bulky hydroxymethyl in selpercatinib than the corresponding methyl group in pralsetinib (Fig. 1c). Thus, differences in the molecular shapes of pralsetinib and selpercatinib were correlated with differences in steric clashes introduced by mutations at sites 730 and 810.

To determine whether the RET L730V/I mutants conferred pralsetinib-specific resistance in vivo, we tested pralsetinib and selpercatinib in BaF3/KIF5B-RET(L730I) and BaF3/KIF5B-RET(L730V) xenograft tumors in hairless SCID (SHO) mice (Supplementary Fig. 3). Similar to that in cell culture (Supplementary Fig. 1c), the BaF3/KIF5B-RET(L730I) tumors grew slightly slower than the BaF3/KIF5B-RET(L730V) tumors (Fig. 1d, h). Previous studies showed that pralsetinib and selpercatinib had a comparable potency in vitro and in vivo on RET oncogene-driven tumors in the absence of the roof mutations, and the antitumor dose–response curves reached a plateau between 10 and 30 mg/kg twice daily (BID)^7,8^. Therefore, we used pralsetinib at 40 mg/kg once daily (QD) and selpercatinib at 16 mg/kg BID in the animal experiment to mimic the ratio and dosing schedule of the recommended dosage of pralsetinib (400 mg QD) and selpercatinib (160 mg BID) in adult patients.

Pralsetinib could not inhibit the L730I or the L730V tumor growth (Fig. 1d–f, h–j). In comparison, selpercatinib caused L730I tumor regression and suppressed L730V tumor growth (Fig. 1d–f, h–j). These results indicated that the L730I and L730V tumors were resistant to pralsetinib, but sensitive to selpercatinib. Consistently, tumor tissues from pralsetinib-treated mice had high levels of phosphorylated KIF5B-RET(L730I) and KIF5B-RET(L730V), and active ERK1/2 as in vehicle-treated mice. In contrast, tumor tissues from selpercatinib-treated mice had greatly reduced levels of these activated kinases (Fig. 1g, k, Supplementary Fig. 4), indicating that KIF5B-RET(L730I) and KIF5B-RET(L730V) kinases were inhibited by selpercatinib, but not by pralsetinib, in the xenograft tumors.

Clinical experience in kinase-targeted cancer therapy has repeatedly shown that acquired resistance will develop and secondary drugs are needed to extend the duration of response. Both pralsetinib and selpercatinib have excellent clinical activity in RET-altered cancers conferring deep and durable responses leading to their FDA approval. Previously identified selpercatinib-resistant RET mutations were also resistant to pralsetinib, making it necessary to develop the next generation of drugs to inhibiting these mutants.

Here, we identified L730V/I mutations at the roof of the solvent front of the RET ATP-binding site as strong pralsetinib-resistant mutations. Recently, using a cell-free DNA (cfDNA) assay that covered the entire coding region of RET, L730V and T729_L730insL were found as on-target acquired mutations in cfDNA of RET fusion-positive NSCLC patients who developed resistance to pralsetinib^15^. Thus, mutations at L730 are clinically relevant mechanisms of pralsetinib resistance. Significantly, unlike G810 mutations located at the floor of the solvent-front site that shared resistance to both selpercatinib and pralsetinib, we showed here that the pralsetinib-resistant L730V/I mutations remained sensitive to selpercatinib. This finding provides an important insight into managing pralsetinib resistance associated with RET L730V/I mutations. Thus, our study suggests that selpercatinib could be an option when pralsetinib-treated cancers progress with L730V/I-positive cfDNA or tumor biopsy for continuing suppression of the RET-altered tumors. On a practical level, this pre-clinical finding may allow clinicians to personalize RET-targeted strategies based upon the emergence of this specific mutation, which may ultimately translate into improved patient outcomes. Further studies to identify cross-resistance profiles of RET inhibitors are warranted.

## Methods

### Isolation and characterization of pralsetinib-resistant RET mutations

A pool of randomly mutated KIF5B-RET cDNA was generated in a lentiviral plasmid using XL1-Red cells and expressed in BaF3 cells by infecting these cells with lentiviruses generated from the randomly mutated plasmid pool as described^12^. Puromycin-resistant, IL-3-independent cells were selected in semi-solid medium in PRMI-1640/10% fetal bovine serum (FBS) with 120, 150, or 300 nM pralsetinib (Table 1). Drug-resistant cell colonies were isolated, purified, and expanded in PRMI-1640/10% FBS containing pralsetinib. Genomic DNA was prepared from individual cell lines, and both strands of the RET kinase domain in the KIF5B-RET cDNA were sequenced to identify mutations. The PCR primers were KR1700 (5′-TTGCTGTGGGAAATAATGATGTAA)/KR2690R (5′-GAGAGGCCGTCGTCATAAATCAGG) or KR1700/KR2295 (5′-CTCTTCATAAACATCTCGGGACA). The sequencing primers were KR1700, KR2272R (5′-AGCCGAAATCCGAAATCTT), KR2295R, or KR2671R (5′-TCAGGGAGTCAGATGGAGTG). Cell proliferation and IC_50_s were determined using CellTiter-Glo reagent (Promega) in 96-well plates after three days of drug treatment as described^12,16^. Immunoblotting assays were performed as described^12^. All gels and immunoblots for each panel were derived from the same experiment, and they were processed in parallel.

### Xenograft tumor assay

The experiment was approved by the Institutional Animal Care and Use Committee of the University of Oklahoma Health Sciences Center. BaF3/KIF5B-RET(L730I) and BaF3/KIF5B-RET(L730V) cells were inoculated s.c. (1 × 10^7^ cells/0.1 ml/each) into the right flank of 5-week old female SHO mice (Charles River). After tumor formation (Fig. 1d, h), mice were divided into three groups (6 mice/group) and treated with pralsetinib (40 mg/kg, QD), selpercatinib (16 mg/kg, BID), or vehicle by oral gavage. Tumor size and animal body weight were measured with a caliper as described^17^. Tumor volume was estimated using the formula *V* = (*L* × *W*^2^)/2, where *V* is the tumor volume, *L* is the tumor length, and *W* is the tumor width. Statistical analysis was performed using the unpaired Mann–Whitney test.

### Reagents

Selpercatinib and pralsetinib were from Chemietek (Indianapolis, IN, USA). Antibodies to phospho-RET(Tyr905) (#3221, 1:1000 dilution), RET (#14698, 1:1000 dilution), phospho-ERK1/2 (#4376, 1:1000 dilution), and cleaved-PARP (#9541, 1:1000 dilution) were from Cell Signaling Technology (Danvers, MA, USA). Anti-total ERK1/2 antibody (#SC-514302, 1:500 dilution) was from Santa Cruz Biotech. Anti-Flag antibody (#F1804, 1:1000 dilution), and anti-β-actin antibody (#A5316, 1:80000 dilution) was from Sigma.

### Reporting summary

Further information on research design is available in the Nature Research Reporting Summary linked to this article.

## Supplementary information

Supplementary Information

Reporting Summary

## Data Availability

The data that support the findings of this study are available from the corresponding author upon reasonable request.
