# Supplementary material for: The L730V/I RET roof mutations display different activities toward pralsetinib and selpercatinib
Source: NPJ Precis Oncol. 2021 Jun 7;5:48. doi: 10.1038/s41698-021-00188-x (PMC8184971; doi:10.1038/s41698-021-00188-x)
Supplement: Supplementary file 1 — Supplementary Information [file 41698_2021_188_MOESM1_ESM.pdf]

SUPPLEMENTARY MATERIALS

The L730V/I RET roof mutations display different activities towards pralsetinib and selpercatinib

Tao Shen[1,2,+], Xueqing Hu[1,2,+], Xuan Liu[1,2,+], Vivek Subbiah[3], Blaine H.M. Mooers[1,4,5], and Jie Wu[1,2,*]

[1]Peggy and Charles Stephenson Cancer Center, [2]Department of Pathology, [4]Department of Biochemistry and Molecular Biology, [5]Laboratory of Biomolecular Structure and Function, University of Oklahoma Health Sciences Center, Oklahoma City, OK 73104

[3]Department of Investigational Cancer Therapeutics, Division of Cancer Medicine, the University of Texas MD Anderson Cancer Center, Houston, TX 77030

[+]These authors contributed equally

*Correspondence to: Dr. Jie Wu, University of Oklahoma Health Sciences Center, 975 NE 10th Street, BRC-413, Oklahoma City, OK 73104, USA, Tel. 405-271-8001 ext. 31092; email: jie-wu@ouhsc.edu

SUPPLEMENTARY FIGURES

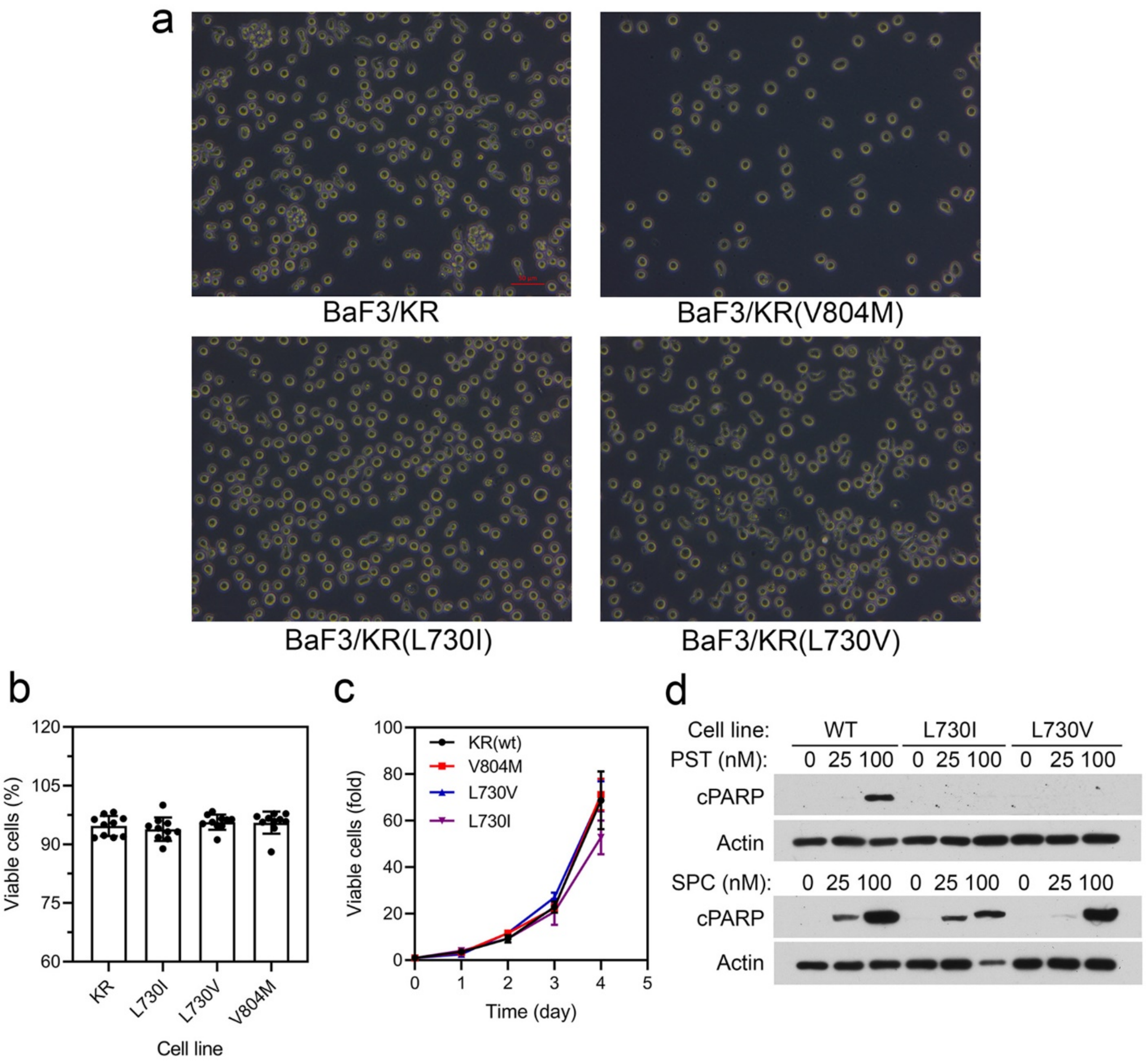

Supplementary Figure 1. Properties of BaF3 cells expressing KIF5B-RET and KIF5B-RET with V804M, L730I, and L730V mutants. a Morphology of cell lines in cell culture plates at 200x magnification. **b** Cell viability determined by Trypan blue exclusion assay. The data were from 3 independent assays. **c** Comparison of cell growth rates. Cells (250 cells/each) were plated in 96-well plates (n=6) and cultured for 0-4 days. Viable cells were measured using the Celltiter glo reagent (Promega). The data were from two independent experiments. On Day 4, the relative number of viable L730I cells was 75% of that of L730V ($p<0.05$), whereas there was not significant difference in the growth rates among the wildtype KIF5B-RET, the V804M, and L730V mutant cell lines. **d** A repeated experiment of immunoblotting analysis of cleaved PARP as that shown in Fig. 1b.

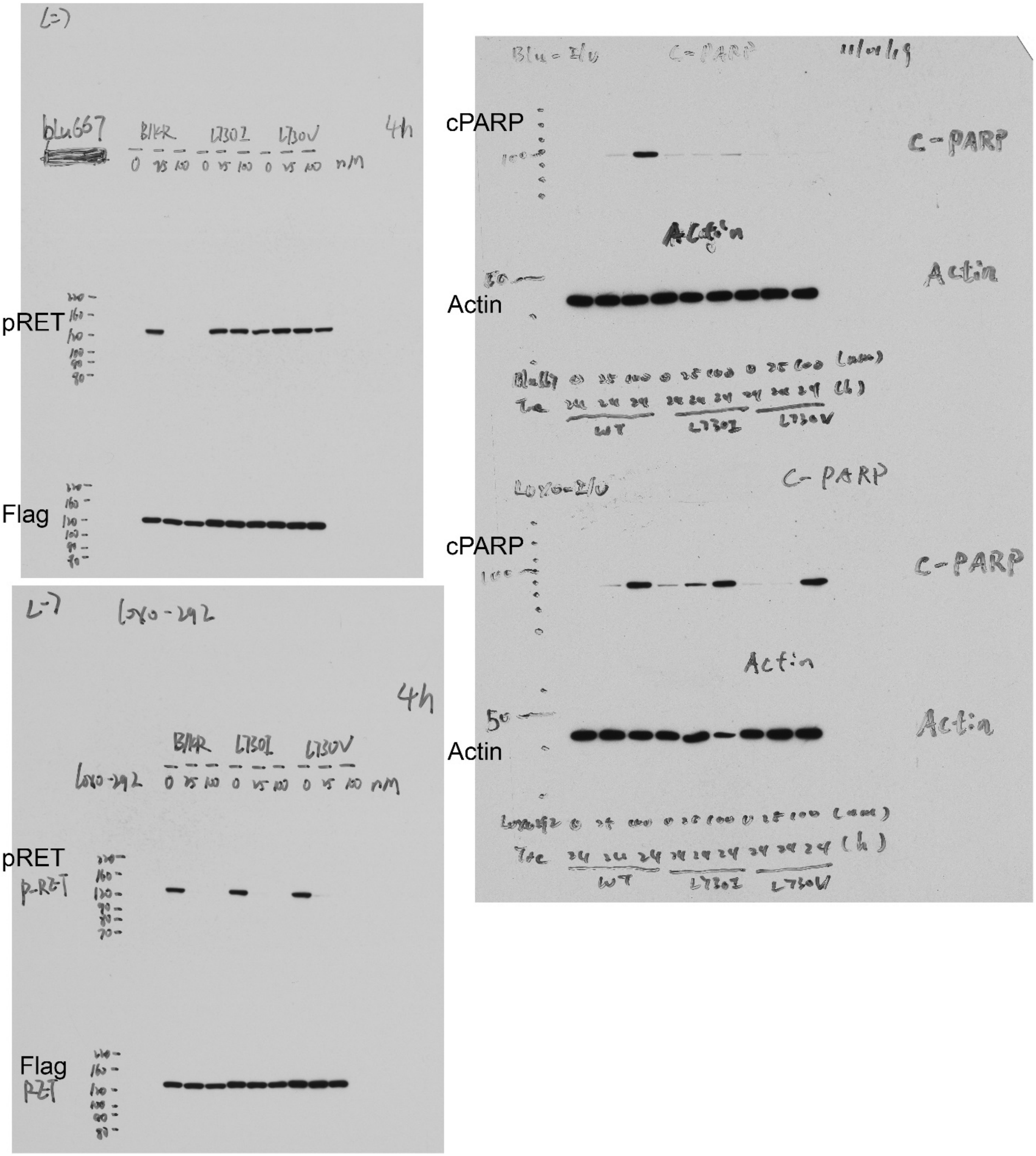

Supplementary Figure 2. Original X-ray films of immunoblots for Fig. 1b. The original scan images of the X-ray films of immunoblots are shown.

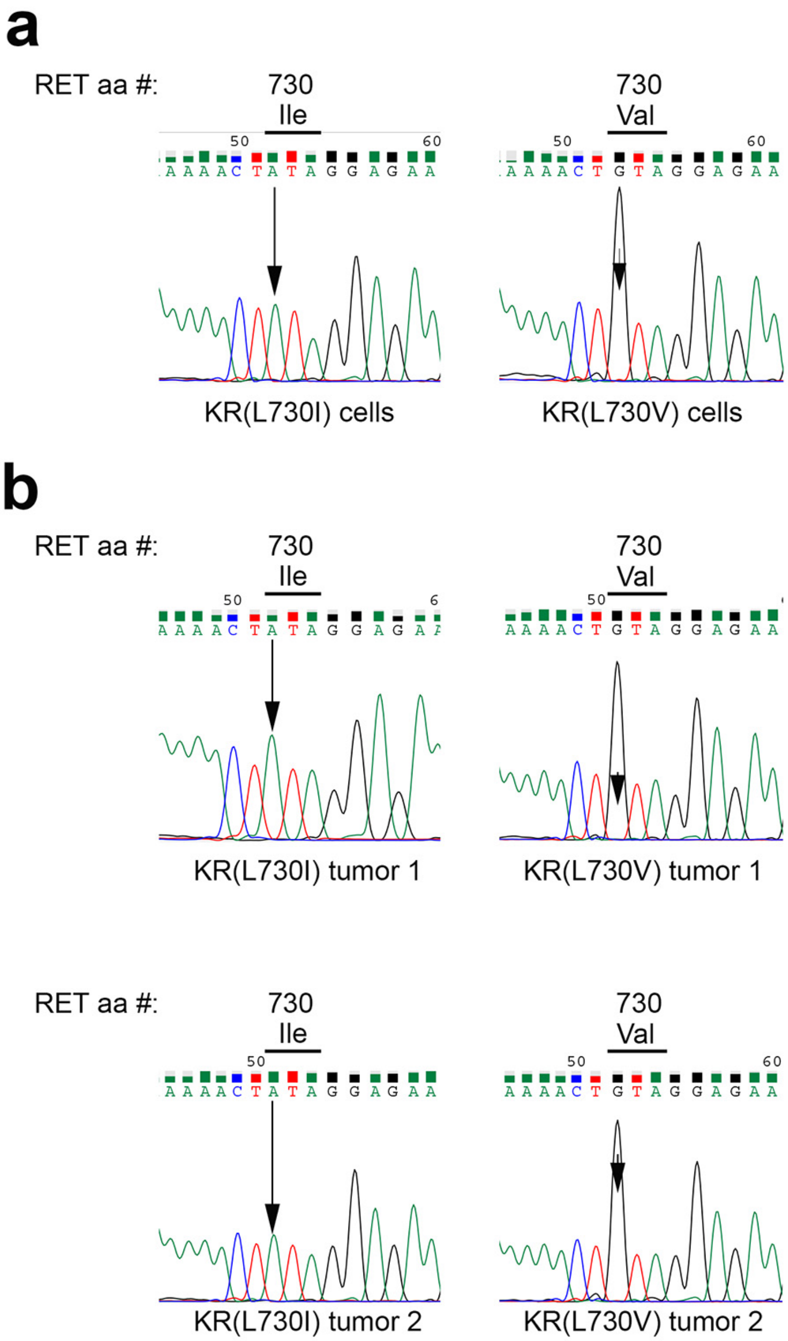

Supplementary Figure 3. DNA sequencing of BaF3/KIF5B-RET(L730I) and BaF3/KIF5B-RET(L730V) cells and tumors. Traces of Sanger DNA sequencing across the L730I or L730V region of each sample are shown. Arrow indicates the mutated residue. **a** DNA samples from BaF3/KIF5B-RET(L730I) cells (left) or BaF3/KIF5B-RET(L730V) cells (right). **b** DNA samples from two BaF3/KIF5B-RET(L730I) tumors (left) or two BaF3/KIF5B-RET(L730V) tumors (right). The tumors were from vehicle-treated groups.

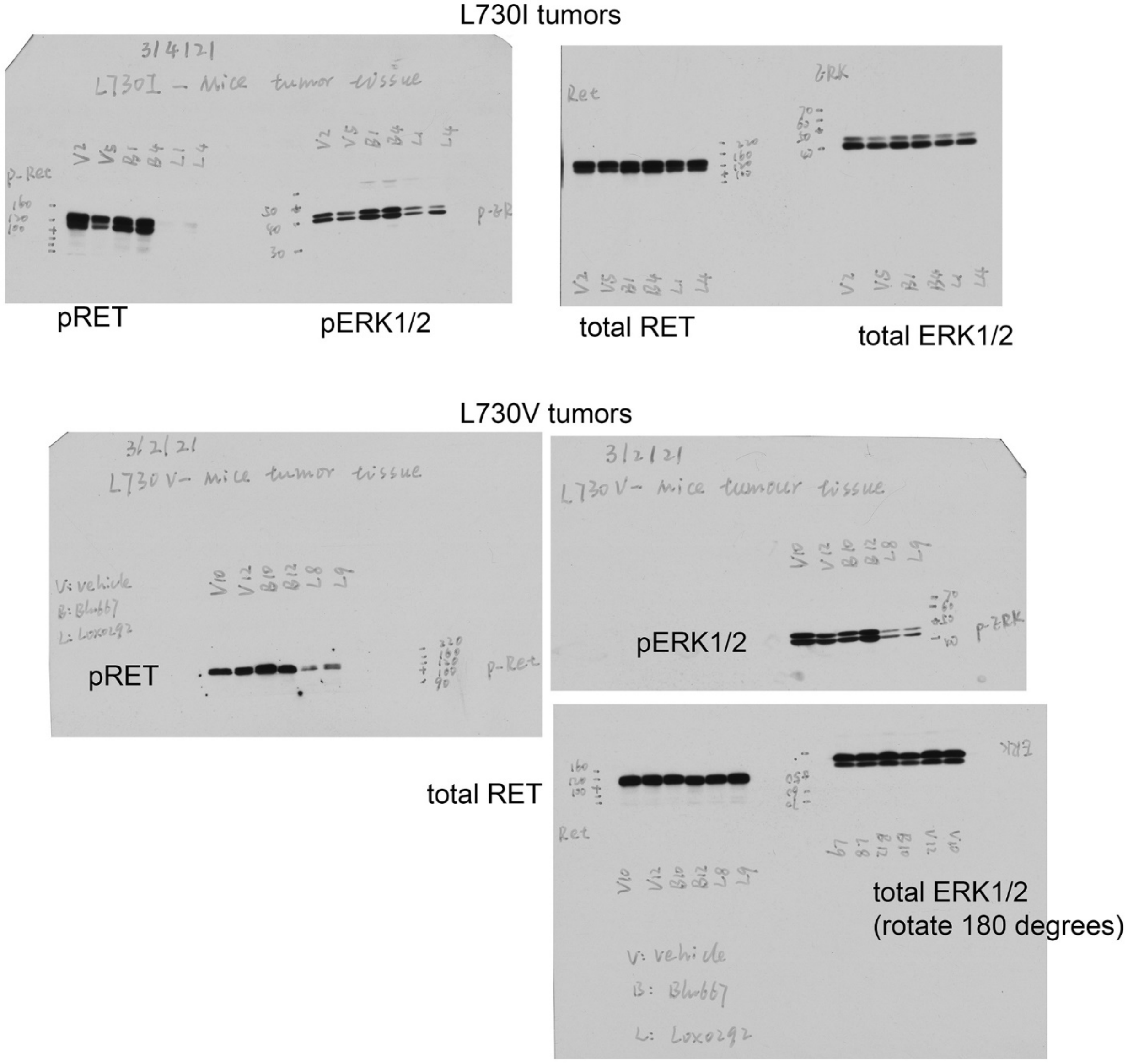

Supplementary Figure. 4. Original X-ray films of immunoblots for Fig. 1g, k. The original scan images of the X-ray films of immunoblots are shown. The samples were renamed as X1 and X2 (X = V, B, or L) in Fig. 1g, k.
