# Supplementary material for: The L730V/I RET roof mutations display different activities toward pralsetinib and selpercatinib
Source: NPJ Precis Oncol. 2021 Jun 7;5:48. doi: 10.1038/s41698-021-00188-x (PMC8184971; doi:10.1038/s41698-021-00188-x)
Supplement: Supplementary file 2 — Reporting Summary [file 41698_2021_188_MOESM2_ESM.pdf]

nature research

Corresponding author(s): NPJPRECISIONONCOLOGY-00928R (Jie Wu)

Last updated by author(s): Apr 14, 2021

Reporting Summary

Nature Research wishes to improve the reproducibility of the work that we publish. This form provides structure for consistency and transparency in reporting. For further information on Nature Research policies, see our Editorial Policies and the Editorial Policy Checklist.

Statistics

For all statistical analyses, confirm that the following items are present in the figure legend, table legend, main text, or Methods section.

n/a	Confirmed	
☐	☒	The exact sample size (n) for each experimental group/condition, given as a discrete number and unit of measurement
☐	☒	A statement on whether measurements were taken from distinct samples or whether the same sample was measured repeatedly
☐	☒	The statistical test(s) used AND whether they are one- or two-sided<br>*Only common tests should be described solely by name; describe more complex techniques in the Methods section.*
☒	☐	A description of all covariates tested
☒	☐	A description of any assumptions or corrections, such as tests of normality and adjustment for multiple comparisons
☐	☒	A full description of the statistical parameters including central tendency (e.g. means) or other basic estimates (e.g. regression coefficient) AND variation (e.g. standard deviation) or associated estimates of uncertainty (e.g. confidence intervals)
☐	☒	For null hypothesis testing, the test statistic (e.g. F, t, r) with confidence intervals, effect sizes, degrees of freedom and P value noted<br>*Give P values as exact values whenever suitable.*
☒	☐	For Bayesian analysis, information on the choice of priors and Markov chain Monte Carlo settings
☒	☐	For hierarchical and complex designs, identification of the appropriate level for tests and full reporting of outcomes
☒	☐	Estimates of effect sizes (e.g. Cohen's d, Pearson's r), indicating how they were calculated

Our web collection on statistics for biologists contains articles on many of the points above.

Software and code

Policy information about availability of computer code

Data collection	n/a
Data analysis	n/a

For manuscripts utilizing custom algorithms or software that are central to the research but not yet described in published literature, software must be made available to editors and reviewers. We strongly encourage code deposition in a community repository (e.g. GitHub). See the Nature Research guidelines for submitting code & software for further information.

Data

Policy information about availability of data

All manuscripts must include a data availability statement. This statement should provide the following information, where applicable:

- Accession codes, unique identifiers, or web links for publicly available datasets
- A list of figures that have associated raw data
- A description of any restrictions on data availability

The data that support the findings of this study are available from the corresponding author upon reasonable request.

Field-specific reporting

Please select the one below that is the best fit for your research. If you are not sure, read the appropriate sections before making your selection.

☒ Life sciences ☐ Behavioural & social sciences ☐ Ecological, evolutionary & environmental sciences

For a reference copy of the document with all sections, see nature.com/documents/nr-reporting-summary-flat.pdf

Life sciences study design

All studies must disclose on these points even when the disclosure is negative.

Sample size	The sample size of 6 in the animal study was based on the data from a previous experiment.
Data exclusions	No data was excluded.
Replication	Cell-based IC50 assays: data were from two or more independent experiments performed in triplicates.<br>Animal-based experiment: tumor sizes were measured during the time course and the weights of excised tumors at the endpoint were measured to confirm the differences in tumor size.
Randomization	Animals were grouped at the beginning of the experiment. No reassignment of individual animals with different tumor sizes at the start of drug treatment.
Blinding	Investigators were not blinded with animals or tumors. Animal groups were labelled on the cage cards and tumor samples were labelled on the tubes.

Reporting for specific materials, systems and methods

We require information from authors about some types of materials, experimental systems and methods used in many studies. Here, indicate whether each material, system or method listed is relevant to your study. If you are not sure if a list item applies to your research, read the appropriate section before selecting a response.

Materials & experimental systems

n/a	Involved in the study	
☐	☒	Antibodies
☐	☒	Eukaryotic cell lines
☒	☐	Palaeontology and archaeology
☐	☒	Animals and other organisms
☒	☐	Human research participants
☒	☐	Clinical data
☒	☐	Dual use research of concern

Methods

n/a	Involved in the study	
☒	☐	ChIP-seq
☒	☐	Flow cytometry
☒	☐	MRI-based neuroimaging

Antibodies

Antibodies used	Information is provided in the main text under the Reagents section.
Validation	Antibodies were verified by immunoblotting analyses of cell lysates of BaF3 cells and BaF3 cells expressing Flag-tagged KIF5B-RET with or without RET tyrosine kinase inhibitors.

Eukaryotic cell lines

Policy information about cell lines

Cell line source(s)	The cell lines were generated in our laboratory from BaF3 cells.
Authentication	BaF3 cells were authenticated by functional assayed for their IL-3-dependent properties , puromycin-sensitivity, and their morphology.
Mycoplasma contamination	Mycoplasm contamination was monitored routinely and within 3 days of injection into animals.
Commonly misidentified lines<br>(See ICLAC register)	N/A

Animals and other organisms

Policy information about studies involving animals; ARRIVE guidelines recommended for reporting animal research

Laboratory animals	Female SHO mice from Charles Rivers, age 5-6 weeks old at the beginning of the experiment.
Wild animals	The study did not involved wild animals.
Field-collected samples	n/a
Ethics oversight	University of Oklahoma Health Sciences Center IACUC.

Note that full information on the approval of the study protocol must also be provided in the manuscript.
